# The Dual-Specificity Phosphatase 10 (DUSP10): Its Role in Cancer, Inflammation, and Immunity

**DOI:** 10.3390/ijms20071626

**Published:** 2019-04-01

**Authors:** Marta Jiménez-Martínez, Konstantinos Stamatakis, Manuel Fresno

**Affiliations:** 1 Department of Cell Biology and Immunology, Centro de Biología Molecular ‘Severo Ochoa’ (CSIC-UAM), 28049 Madrid, Spain; mjimenez@cbm.csic.es (M.J.-M.); kstamatakis@cbm.csic.es (K.S.); 2 Department of Molecular Biology, Universidad Autónoma de Madrid, 28049 Madrid, Spain; 3 Instituto de Investigación Sanitaria la Princesa (IIS-P), 28006 Madrid, Spain

**Keywords:** DUSP10, MAPK, inflammation, cancer

## Abstract

Cancer is one of the most diagnosed diseases in developed countries. Inflammation is a common response to different stress situations including cancer and infection. In those processes, the family of mitogen-activated protein kinases (MAPKs) has an important role regulating cytokine secretion, proliferation, survival, and apoptosis, among others. MAPKs regulate a large number of extracellular signals upon a variety of physiological as well as pathological conditions. MAPKs activation is tightly regulated by phosphorylation/dephosphorylation events. In this regard, the dual-specificity phosphatase 10 (DUSP10) has been described as a MAPK phosphatase that negatively regulates p38 MAPK and c-Jun N-terminal kinase (JNK) in several cellular types and tissues. Several studies have proposed that extracellular signal-regulated kinase (ERK) can be also modulated by DUSP10. This suggests a complex role of DUSP10 on MAPKs regulation and, in consequence, its impact in a wide variety of responses involved in both cancer and inflammation. Here, we review DUSP10 function in cancerous and immune cells and studies in both mouse models and patients that establish a clear role of DUSP10 in different processes such as inflammation, immunity, and cancer.

## 1. Introduction

The human genome contains a large number of genes that transcribe/translate to four families of protein tyrosine phosphatases (PTP), which are subclassified depending of their substrate, structure, regulation, and function. Of these genes, 61 encode for dual-specificity phosphatase subfamily and 10 of them are catalytically active MAP kinase phosphatases (MKPs), which are able to dephosphorylate dual specificity phospho-Tyrosine (pTyr) and phospho-Threonine (pThr) substrate, and one of them is catalytically inactive MKP [1]. DUSP10, also called MKP5, is a member of the MKPs subfamily involved in cell proliferation, differentiation, and migration [2]. Different studies have described a role for DUSP10 as a negative regulator of p38 and c-Jun N-terminal kinase (JNK) through their dephosphorylation [3]. However, new studies confer to DUSP10 the ability to regulate ERK1/2 activity [4]. Nonetheless, it cannot be discarded that the DUSP10 may regulate other proteins either through physical interaction or/and by its phosphatase activity. Up-to-date, enhanced expression of DUSP10 has been reported in several malignancies such as colon, prostate, and breast cancer and diseases such as multiple sclerosis, atherosclerosis, diabetes, celiac disease, and asthma. In addition, elevated DUSP10 expression is relevant to innate and adaptive responses by reducing an excessive inflammatory response. Here, we report an overview of DUSP10 function in different tumors and inflammatory diseases. These results show that DUSP10 is over-expressed in several major cancers, upregulated by numerous physiological stimuli and chemical compounds, and able to control the inflammatory response. The available data suggests that DUSP10 is a potent pro-tumorigenic and anti-inflammatory gene and therefore represents an attractive therapeutic target for the treatment of some cancers and inflammatory diseases.

## 2. Characteristics, Structure, and Function of DUSP10

**Figure 1:**
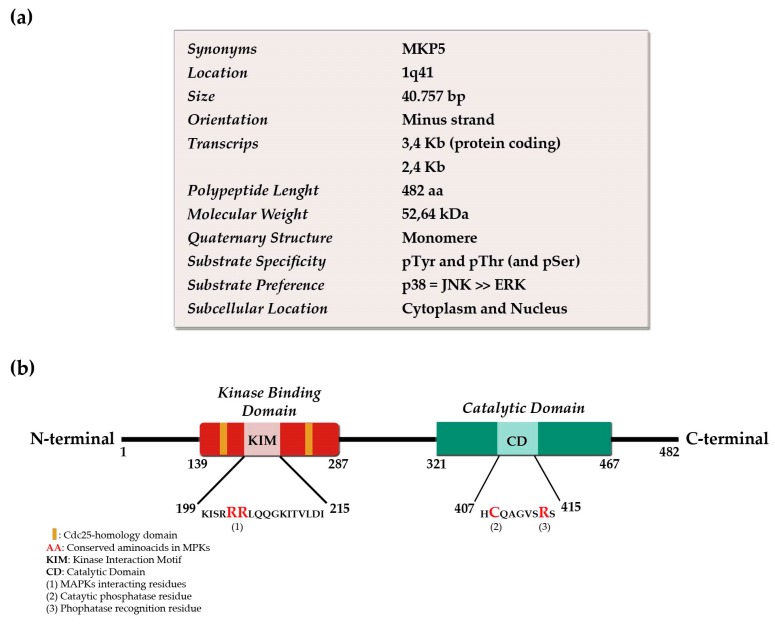
Characteristics and domain organization of human *DUSP10.* (**a**) Summary of the characteristics and information about the gene and protein; (**b**) domain organization of the protein and the most important and conserved polypeptide sequences for the phosphatase activity and interaction capacity.

Two transcripts of approximately 3.4 and 2.4 Kb have been identified within human genome *DUSP10* sequence. The long transcript is widely expressed in human tissues such as skeletal muscle and liver, and its expression is elevated by stress stimuli in cell culture [5]. In most mouse tissues, a 3.5 Kb transcript was highly expressed. However, the 2.7 Kb mRNA splice variant is specifically expressed in both mouse and rat testis [6] (Figure 1a). DUSP10 protein has two Cdc25 homology regions, a C-terminal catalytic domain and a particular 150 N-terminal amino acid sequence with unknown function, that differs from other family members [7] (Figure 1b).

The crystal structure characterization of human DUSP10 catalytic and kinase-binding domains was decisive to define its affinity for substrates. The MAP kinase-binding domain determines a main structural difference from other MPKs, which have little effect on p38 and JNK dephosphorylation. DUSP10 catalytic domain is particular because of an active conformation structure on its own [8,9]. The phosphorylation state of substrate is also important for DUSP10 affinity. For example, bi-phosphorylated p38α in Tyr-182 and Thr-180 is a more efficient substrate than mono-phosphorylated p38α, with pTyr being dephosphorylated slightly faster by DUSP10 than pThr [10]. Similarly, the crystal structure of p38α reveals a particular mode of interaction between the p38α docking site and the kinase-binding domain of DUSP10, which conserves the mechanism of molecular recognition in p38 [11,12]. Concomitantly, JNK1 interacts with DUSP10 using a different substrate-recognition mechanism than p38α through the catalytic domain, but not the kinase-binding domain of DUSP10 [13].

DUSP10 is a dual specificity phosphatase capable of dephosphorylating both pTyr and pThr residues of activated MAPKs with different affinities. It has been shown that isoforms of the p38 and JNK subfamilies are selectively and more effectively dephosphorylated than ERK subfamily members [7]. Additionally, it has been demonstrated that DUSP10 is able to remove phospho-residues and regulate ERK2 activity, with a *Km* more than 100-fold lower than a specific ERK phosphatase such as MKP3 [14]. Another study has shown that DUSP10 may act as a scaffold protein of ERK. DUSP10 negatively regulates ERK by retaining it in the cytoplasm, avoiding ERK enzymatic activity and downregulating the transcription of ERK-dependent genes [4]. However, current studies have also demonstrated that DUSP10 is able to dephosphorylate phospho-Serine (pSer) residue of non-MAPK substrates [15].

## 3. Expression and Regulation of DUSP10

DUSP10 mRNA expression has been detected in almost all tissues, but generally at low levels, except in liver and hematopoietic systems [5,16]. Our in silico UCSC Xena database analysis of available RNA-seq and microarray expression databases has confirmed that myeloid and T cells have the highest expression of DUSP10 [17]. Very few studies have addressed the transcriptional regulation of DUSP10 gene. The GTRD database analysis indicates (http://gtrd.biouml.org) (access on 6 January 2019) that DUSP10 mRNA transcription may be regulated by several transcription factors such as PPAR, EGR, STAT5b, RUNX1, VDR, NFAT5, and NF-κB, among others [18]. Several chromatin immunoprecipitation-mass sequencing (CHIP-seq) experiments reveal the binding of these factors on regulatory elements of DUSP10 gene (promoter region, as identified by H3K4me3 binding) [19]. In this regard, it has been proposed that DUSP10 gene has a particularity of being regulated by MAPKs, which promote DUSP10 expression, suggesting a negative feedback loop [20]. 

**Figure 2:**
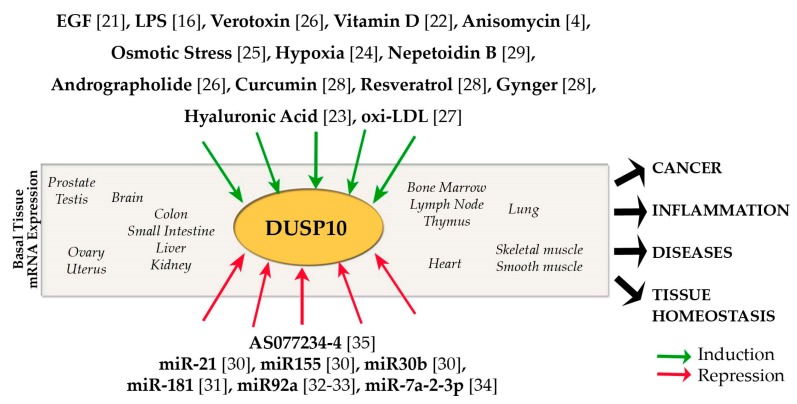
The effect of different signals on *DUSP10* mRNA expression affecting the development of cancer and diseases, controlling inflammatory responses, and altering tissue homeostasis. *DUSP10* mRNA is basally expressed in a wide variety of tissues and it is up- or downregulated by stimuli and inhibitors such as pharmacological agents or miRNAs.

Described stimuli that trigger DUSP10 expression are summarized in Figure 2. Among them, epidermal growth factor (EGF) [21], vitamin D [22], and hyaluronic acid [23] can be found. Increases in DUSP10 have been also observed in hypoxia [24], and in response to some stresses such as anisomycin [4] or osmotic pressure [25]. Interestingly, several innate immunity receptor ligands such as pro-inflammatory LPS [16], verotoxin [26], and oxi-LDL [27] induce DUSP10 expression in macrophages. Surprisingly, a few anti-inflammatory compounds such as resveratrol, ginger, curcumin [28], andrographolide [26], and nepetoidin B [29] also increase DUSP10, suggesting that some of their anti-inflammatory activity can be mediated through DUSP10 suppression of MAPK pro-inflammatory activities. Although most studies have shown that LPS induces DUSP10 expression, a few (i.e., Reference [29]) show the contrary. Those differences may be ascribed to the different cell types used. It has also been shown that cytotoxic *Shigella* toxin (Stx1 or Verotoxin) induces DUSP10 expression as effectively as lipopolysaccharide (LPS) and up-regulates other genes such as cyclooxygenase-2 (*COX-2*) and early-growth response protein 1 (*EGR-1*) [26].

On the other hand, different groups have described how the DUSP10 gene is negatively regulated by mirRNAs, which are induced in different diseases and cancers. *DUSP10* is a direct target for miR-21, miR-30b, and miR-155, and has a specific binding site sequence in its 3′-untranslated region where this gene may be negatively regulated. A negative feedback loop in the regulation of *DUSP10* by miRNAs, may exist, since miR-21 and miR-155 transcription is dependent of AP-1 activity, a transcription factor downregulated by *DUSP10* expression [30]. In hepatocellular cancer and pancreatic cancer, miR-181 and miR92a, respectively, negatively regulate the DUSP10 expression, affecting the proliferation and migration of tumorigenic cells [31,32]. Enhanced mir-92a expression is also detected at the peak of the EAE and downregulates *DUSP10* transcription [33]. In hypoxic-ischemic brain damage, *DUSP10* is a target downregulated by miR-7a-2-3p in rats [34]. 

Only one pharmacological inhibitor (AS077234-4) has been described for DUSP10 to date. However, the information about this inhibitor and its exact mechanism of action is scarce [35].

## 4. DUSP10 in Inflammation and Immunity

**Figure 3:**
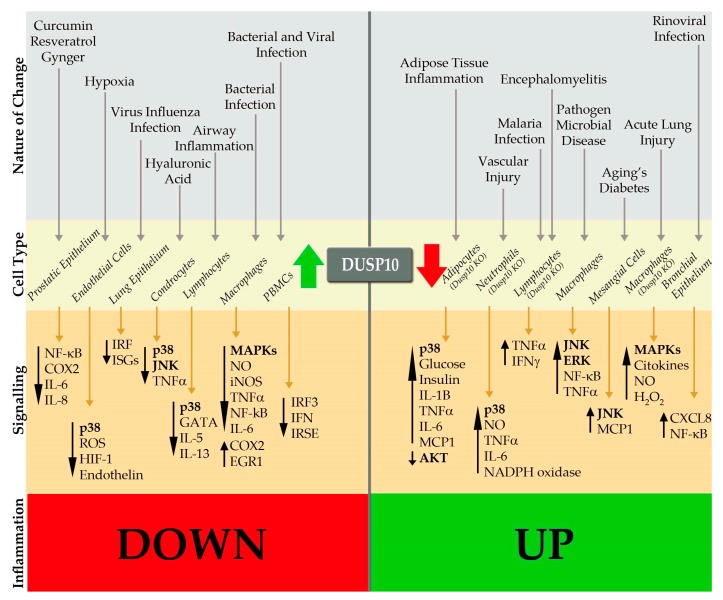
Summary of the role of DUSP10 in response to different infection stimuli and disease conditions. Depending on the nature of the insult/stimulus, DUSP10 expression can be up- (green arrow) or downregulated (red arrow) in each specific cell type. In consequence, it negatively or positively regulates different signal transduction cascades and effector molecules such as mitogen-activated protein kinases (MAPKs), cytokines, interleukins, etc., promoting down- or up-control of inflammation.

Zhang et al. first identified DUSP10 as an inducible phosphatase during immune responses. In naïve mouse lymphocytes, *Dusp10* gene is constitutively expressed, but downregulated following T-cell receptor activation. In contrast, *Dusp10* is not constitutively expressed in macrophages and upregulated after LPS stimulation. They described that this phosphatase was not needed for development of the immune system, while is required in innate and adaptive immune responses, limiting p38 and JNK activity and regulating cytokine production [16]. Following this, several groups have focused on the activity of DUSP10 in immunity and inflammation in different disease settings. Thus, after infection, injury, disease induction, or treatment with several stimuli, DUSP10 can be upregulated or downregulated resulting in different cellular and molecular outcomes, leading to decreased or increased inflammation respectively (Figure 3).

A number of “natural” anti-inflammatory agents have been identified as able to upregulate DUSP10 expression, which in turn regulate inflammatory mediators through MAPKs. The treatment with Andrographolide (*Andrographis paniculata*) in endothelial cells induces DUSP10 expression, reducing hypoxia-induced HIF-1α expression and endothelin-1 secretion through decreased p38 activity [24]. Another compound is Nepetoidin B, which induces DUSP10 expression in macrophages, repressing LPS-induced nitric oxide (NO) production with a p38- and JNK-dependent effect, but no ERK [29]. Hyaluronic acid (HA) also induces DUSP10 expression, negatively regulating p38 and JNK activation by TNF-α in chondrocytes and promoting anti-inflammatory response in osteoarthritis [23].

As mentioned above, DUSP10 is able to dephosphorylate JNK or p38, but some reports have also described a role of DUSP10 through ERK and even dephosphorylation of some other non-MAPK targets. Initially, its effects on T-cells and macrophages were ascribed mostly to JNK deactivation [36]. However, DUSP10 negatively modulates p38 MAPK activity in macrophages that leads to downregulate inflammatory mediators such as TNF-α and IL-6 [37]. The *Shigella* toxin-induced TNF-α expression is reduced by inhibition of ERK in the human macrophage cell line THP-1. This shows the important role of MAPKs in inflammation and the necessity of regulating these pathways [26]. In response to infection of THP-1 cells with *Porphyromonas gingivalis*, a common pathogen associated with chronic adult periodontal disease, DUSP10 expression is decreased. Overexpression of DUSP10 in THP-1 cells reduced activation of ERK and JNK and, thus cytokine production, in response to *P. gingivalis* [20].

Manley et al. show that rhinoviral infection of airway epithelial cells downregulates DUSP10 expression leading to increased activation of p38 and JNK. Reduced DUSP10 levels potentiate the production of CXCL8 and CXCL1 but not IFN-β production in response to infection or IL-1β alone [38]. This suggests that decreases in DUSP10 lead to inflammation in airway diseases such as asthma, likely through regulating inflammasome activation [38]. ASC protein, a downstream common adaptor for different proteins within the inflammasome complex, induces chemokine expression through modulating ERK and JNK activation and, in addition, downregulates DUSP10 expression in THP-1 cells and mouse macrophages [20]. In contrast, a different virus infection (Influenza) induces DUSP10, reducing IRF3 nuclear accumulation that leads to downregulating type I IFN expression and T cell response in the lung epithelial cells. Interestingly, James et al. demonstrated that DUSP10 is able to specifically interact with dephosphorylate IRF3, independently of its interaction with MAPKs. They demonstrated that *Dusp10*-deficient macrophages increase the type I IFN expression and ISGs (IFN-stimulated genes) through IRF3 phosphorylation in response to TLR3 activation upon influenza virus inflection, which reduces its replication in *Dusp10* KO cells and increases innate immune responses [15]. In agreement with this, DUSP10 expression reduces the ISRE (IFN-stimulated response element) activity in the promoter region of RANTES [39]. Together, the above results imply specific roles for DUSP10 in viral infections likely depending on differential TLR signaling by each virus. In allergic airway inflammation, *Dusp10* is highly expressed in specific pathogenic Th2 cells. DUSP10 decreases p38 and GATA3 activity, thus negatively regulating cytokine production in response to IL-33, such as IL-13 and IL-5, and limits the inflammatory response [19]. 

A pro-inflammatory phenotype is observed in aging diabetic mesangial cells by an excessive oxidative stress, where decreased DUSP10 expression promotes a phosphorylated JNK activation and elevated MCP-1 production, which contributes to chronic inflammatory lesions and disease progression [40]. DUSP10 has been also proposed as a target for therapeutic treatment of atherosclerosis, since DUSP10 inhibition reduces NF-κB-induced TNF-α expression and increases TGF-β1 levels in this disease [27]. 

In prostatic cell types, DUSP10 overexpression is associated with a potent anti-inflammatory activity through modulation of p38-dependent signaling. DUSP10 overexpression, acting as ant-inflammatory protein, decreases pro-inflammatory responses mediated by cytokine-dependent NF-κB activation, COX-2 expression, and cytokine (IL-6 and IL-8) production in non-transformed prostatic epithelial cell lines [28]. Induced DUSP10 also decreases IL-6 expression through p38 inactivation after 1-alpha,25-dihydroxyvitamin D_3_ (1,25D) or calcitriol treatment, promoting a decrease of prostatic inflammation [41].

The use of *Dusp10*-deficient mice allowed the elucidation of the role of this phosphatase on the regulation of immunity and inflammation, demonstrating that it plays an essential non-redundant role in regulating some aspects of immune function against infections and other diseases. The deficiency of Dusp10 expression in mice produces a higher vascular inflammatory response, increasing mainly p38 activation, erythrocyte extravasation, microcapillary thrombus formation, and neutrophil accumulation against LPS injection, which help to propose Dusp10 as a negative regulator of activation of neutrophil inflammatory response against injury by negatively regulating NADPH oxidase [42]. Deficient Dusp10 expression in a mouse model of sepsis-induced lung injury has also been involved in the positive production of pro-inflammatory cytokines such as IL-6, TNF-α, MIP-2, NO, and ROS levels in lung alveolar macrophages and promoting the resistance of mice to LPS-induced sepsis in acute lung injury [43]. DUSP10 is also implicated in metabolic regulation of adipose tissue inflammation. Dusp10-deficient mice develop adiposity, insulin resistance spontaneously, and glucose intolerance. Macrophages from Dusp10 deficient mice are activated, polarized to an M1 state, and infiltrate white adipose tissue, which shows enhanced p38 activity and reduced AKT activation [44].

Dusp10-deficient mice are resistant to experimental myelin-induced autoimmune encephalitis (EAE), an animal model of multiple sclerosis. DUSP10 was found to be a negative regulator of both Type 1 (IFN-γ) and Type 2 (TNF-α) cytokine expression in effector CD4 and CD8 T-cells, reducing AP-1 expression through regulating JNK activity. Thus, DUSP10 expression protects the host from injury due to excessive T-cell response to pathogens [16]. It has been shown that CD11c+ dendritic cells from Dusp10-deficient mice have enhanced antigen presentation activity to splenic CD4+ T-cells resulting increased IFN-γ production [45]. Moreover, this enhanced pro-inflammatory response observed in absence of Dusp10 may result beneficial or deleterious to the murine host, depending largely on the pathogenicity of the infecting parasite strain. Thus, DUSP10, is a strong regulator of the host immune response, regulating antigen presentation by both dendritic cells and T-cell responses.

Thus, the available data to date support that the function of this phosphatase is complex and cellular and disease context dependent (Figure 3).

## 5. DUSP10 and Cancer

**Figure 4:**
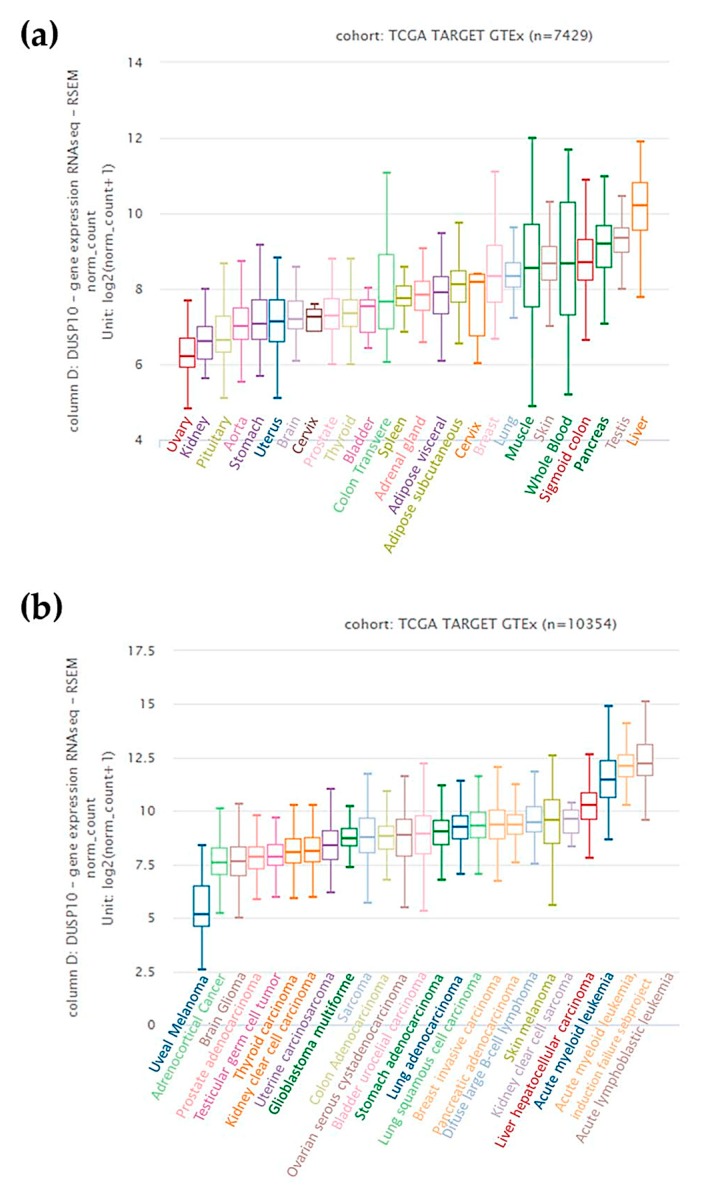
Analysis in silico of *DUSP10* mRNA levels in human samples. (**a**) Normal tissue and (**b**) tumors using the UCSC Xena Browser-GTEx cohort and the TCGA-ARGET cohort, respectively.

There have also been a few reports regarding the putative role of DUSP10 in cancer [46]. Most of those studies have found increased *DUSP10* mRNA in tumor tissue, thus suggesting a pro-tumorigenic role for this phosphatase. Nonetheless, there are some studies assigning a suppressive role of DUSP10 in cancer. The in silico UCSC Xena database (https://xena.ucsc.edu/) (access on 6 January 2019) analysis [17] indicates that basal *DUSP10* mRNA expression of normal tissues is low, except in liver and some blood cells (Figure 4a). However, *DUSP10* in most of the cancerous tissues has higher expression levels than that corresponding to normal tissue, although there is variability depending on the tumor type and even within a tumor type. This is particularly striking in some cancers such as acute myeloid leukemia, acute lymphoblastic leukemia, and, to a lesser extent, in hepatocellular carcinoma. Some types of metastatic melanoma and breast cancer carcinomas also show elevated *DUSP10* expression (Figure 4b). 

It is well known how p38 and JNK MAPKs are implicated in a variety of cell processes such as migration, proliferation, differentiation, and survival. The regulation of this signaling is very important for tumorigenicity. In consequence, it is crucial to regulate this pathway to control cancer progression and to develop therapeutic strategies [47]. A study with mice lacking *Dusp10* demonstrated the role of this phosphatase in muscular dystrophy, a degenerative skeletal muscle disease. In this case, Shi et al. described that Dusp10 is a negative regulator of muscle stem cell function in mice, decreasing cell proliferation and myogenesis by selective p38 and JNK dephosphorylation [48]. 

On the other hand, several research studies in cancer during the last years have reported *DUSP10* upregulation in colon cancerous tissue and to a lesser extent in other cancers such as lung, breast, prostate, and glioblastoma [4]. However, further basic and clinical investigations in specific diseases would be required to determine the role of DUSP10 in cancer. Below, we will summarize main studies about the presence of DUSP10 in cancer reported to date. 

### 5.1. Hepatocellular and Pancreatic Cancer

In human hepatocellular cancer (HC), low expression of p73 (TP53INP1) diminishes its binding as transcription factor to DUSP10 promoter, downregulating the phosphatase expression, and promoting metastasis via increased ERK activation [49]. This is despite higher *Dusp10* mRNA levels observed in these tumors (see Figure 4b). Along similar lines, miR-181 family represses DUSP10 transcription by a direct and sequence-specific manner the miRNA binds to the 3′ UTR of *DUSP10* mRNA. This leads to increased p38 phosphorylation, which promotes HC cell migration [50]. Thus, in HC, low expression of DUSP10 seems to be associated to migration and metastasis. In human pancreatic cancer (PC), JNK activation through DUSP10 downregulation, which is directly targeted by miR-92a, promotes the proliferation of cancerous cells [51]. 

### 5.2. Gastrointestinal Cancer

*DUSP10* mRNA is frequently over-expressed in colon carcinoma and different polymorphisms (SNPs) have been identified near or within the sequence of *DUSP10* gene, which are correlated with colorectal cancer (CRC) risk. In a Chinese population, two SNPs (rs908858 and rs11118838) lead to the haplotypes ‘ACTCAACTA’ or ‘GCCCACCCA’, which correlate with an increased or a decreased CRC risk, respectively [31]. These polymorphisms and SNP rs12724393 are associated with less CRC risk in females of Chinese population; while only the rs908858 is linked with a decreased risk in males [32]. Another SNP (rs6687758) located downstream of DUSP10 is associated with tooth agenesis, which in the family history of cancer positively correlated with a specific haplotype for this polymorphism [52]. Moreover, the last described SNP is a common genetic variant near DUSP10 that increases CRC risk in alcohol consumers in Korean population [53]. These studies support the idea that enhanced DUSP10 is related with CRC risk, and specific polymorphisms that modify its levels could alter the activity of direct DUSP10-targets or other important pathways for the tumour.

In mouse models of intestinal inflammation-associated with dextran-sulphate sodium (DSS) induced colitis, it has been demonstrated that c-GMP-dependent protein kinase (PKG2) induces *Dusp10* expression in colon and damages luminal epithelium and the intestinal immune system, by reducing phospho-JNK and suppressing apoptosis [54]. In apparent contradiction, Png et al. reported that *Dusp10* KO mice were more resistant to DSS-induced colitis by increasing ERK1/2 activation and KLF5 expression [55]. This reduction of colitis eventually led to a decrease in the colon polyp number after azoxymethane (AOM) carcinogen and DSS treatment. In other reports, p53 binding to JNK prevents its dephosphorylation by up-regulating DUSP10 and promoting apoptosis in CRC cell lines upon genotoxic stress [56]. 

### 5.3. Breast Cancer

In the context of breast cancer (BC), MKPs subfamily is differentially expressed depending on the specific subtype of malignancy. However, DUSP10 is an upregulated gene in the major BC studies. For example, DUSP10 has been identified as an induced gene in HER2-positive breast tumors [57]. In agreement with this, microarray phosphatome profiling of BC patients has shown that DUSP10 is over-expressed in a specific BC genetic profile, with both estrogen receptor (ER)-negative and epidermal grown factor receptor 2 overexpressing BC [58]. Another study revealed that ER-positive and p53 wildtype BC patients have a worse overall survival associated to AGR2 over-expression, which directly induces DUSP10 expression [59]. In addition, DUSP10 can be induced by oxidative stresses such as H_2_O_2_ and binds to over-oxidized peroxiredoxin; this complex preserves the DUSP10 activity, leading to p38 inactivation and reducing cancer-associated senescence in BC [60].

### 5.4. Prostate Cancer

In prostate cancer (PC), studies to date have been focused on the relationship between DUSP10 expression with 1,25D, the hormonally active metabolite of vitamin D, used to treat this disease. Thus, it has been identified by cDNA microarray analysis that *DUSP10* expression is increased in primary cultures of human prostatic epithelial normal and cancer cells, but not in normal prostatic stromal cells, after 1,25D treatment [22]. The upregulation of DUSP10 by 1,25D occurs in primary prostatic adenocarcinoma, while DUSP10 expression is absent in PC cell lines derived metastases, leading to pro-carcinogenic inflammation. This *DUSP10* up-regulation is due to a specific vitamin D receptor (VDR) response element within the DUSP10 promoter [41]. That link between them is also observed in human peripheral blood mononuclear cells (PBMCs) from patients, where DUSP10 is induced in response to 1,25D through VDR [61,62]. Besides inducing DUSP10, 1,25D treatment also inhibits prostaglandin synthesis through decreasing COX-2 and prostaglandin E_2_ and F_2α_ receptor (EP and FP) expression and increasing 15-prostaglandin dehydrogenase. Furthermore, the combination of 1,25D and non-steroidal anti-inflammatory drugs (NSAIDs) synergistically reduces PC cell growth, and the authors propose that this pathway is a possible therapeutic approach to PC [28]. In consequence, DUSP10 has an anti-inflammatory effect in PC, although its elevated expression promotes carcinogenesis. However, equilibrium between an anti-inflammatory microenvironment and DUSP10 expression levels could be important to regulate PC.

### 5.5. Other Cancers and Diseases

DUSP10 is expressed in the majority of meningiomas of all grades, regulating cell proliferation and tumor progression. This tumorigenic phenotype is due to DUSP10 dephosphorylates p38, in which inactivation is determinant in the pathogenesis of meningiomas [63]. The use of specific DUSP10 inhibitor (AS077234-4) decreases the DUSP10 transcription and, in consequence, an induction of oligodendrocyte differentiation of cortical precursors is observed. Authors suggest DUSP10 inhibition as a possible target for treating dysfunctional myelin deposition-associated diseases such as multiple sclerosis [35]. Highly expressed DUSP10 is also found in a common bone marrow disease such as acute myeloid leukemia (AML) [64] and its expression correlates with disease subtypes [65]. In fact, our analysis of available databases indicates that AML cells have the highest DUSP10 mRNA expression of all cancer types. The importance of this for AML pathogenesis and treatment remains to be determined and deserve further investigations.

## 6. Conclusions

The literature discussed above reveals important roles for DUSP10 in immunity, inflammation, and cancer. The relationship between cancer and inflammation has been proposed years ago, representing an essential communication of cytokines and immune cells with neoplastic cells that occurs in tumor microenvironment [66]. Several studies have demonstrated how DUSP10 is an important phosphatase expressed in a wide variety of tissues and upon different conditions. We should underline that a variety of stress stimuli such as sepsis, injury, chronic inflammation, and viral infection modulates DUSP10 expression. Depending on the nature of this change, DUSP10 is inhibited or increased and regulates the innate and immune response leading to a pro- or anti-inflammatory activity. A large number of experimental data report that DUSP10 has an anti-inflammatory role. Particularly, DUSP10 is induced in many types of cancers, and its enhanced expression confers in general a pro-tumorigenic capacity to neoplastic cells for proliferation, migration, and differentiation. However, in some cases, DUSP10 inflammation modulating activity may also suppress tumor development in an indirect way in some inflammation-driven cancers pointing our complex role of DUSP10 in cancer. Therefore, investigation into the role of DUSP10 in inflammation in cancer would be of great interest. In addition, the action of DUSP10 on cancer, inflammation, and immunity may take place through inactivation of its ‘classical’ targets p38 and JNK, but also through ERK or by directly targeting other proteins like IRF3 in a MAPK-independent manner. Summarizing, DUSP10 expression has an essential role in controlling tumor development and inflammatory response through MAPKs or other novel and different targets. Moreover, DUSP10 is a good anti-cancer and anti-inflammatory therapeutic target, but more studies are needed to elucidate its role in each specific cellular and physiological context.
